# The Chronic Angioedema Registry: what the first 2 years since the implementation of the global registry have taught us

**DOI:** 10.3389/falgy.2026.1799211

**Published:** 2026-06-22

**Authors:** Thomas Buttgereit, Mathias Sulk, Jonathan A. Bernstein, Alicja Kasperska-Zajac, Andreas Kleinheinz, Ankur Jindal, Anna Valerieva, Danny M. Cohn, Henriette Farkas, Herberto Jose Chong-Neto, Ivan Cherrez-Ojeda, Ivan Markovic, Kanokvalai Kulthanan, Mohamed Abuzakouk, Moshe Ben-Shoshan, Murat Türk, Natasa Teovska Mitrevska, Noemi A. Bara, Oscar Calderon Llosa, Philip H. Li, Ramon Lleonart Bellfill, Renate Krüger, Riccardo Senter, Roman Hakl, Sabine Altrichter, Simon Francis Thomsen, Stefan Cimbollek, Nevenka Adjievska, Lasma Lapina, Jonny Peter

**Affiliations:** 1 Institute of Allergology, Charité – Universitätsmedizin Berlin, corporate member of Freie Universität Berlin, Humboldt-Universität zu Berlin, Berlin, Germany; 2 Fraunhofer Institute for Translational Medicine and Pharmacology ITMP, Immunology and Allergology, Berlin, Germany; 3 Department of Dermatology, University Hospital Münster, Münster, Germany; 4 Department of Internal Medicine, Division of Rheumatology, Allergy and Immunology, University of Cincinnati College of Medicine, Cincinnati, OH, United States; 5 European Center for Diagnosis and Treatment of Urticaria, Medical University of Silesia, Katowice, Poland; 6 Department of Dermatology, Clinical Centre Buxtehude, Buxtehude, Germany; 7 Division of Paediatric Clinical Immunology and Rheumatology, Department of Paediatrics, Manipal Hospitals, Bengaluru, India; 8 Pediatric Allergy Immunology Unit, Department of Pediatrics, Postgraduate Institute of Medical Education and Research, Chandigarh, India; 9 Department of Allergology, Medical University of Sofia, Clinic of Allergology, University Hospital “Alexandrovska”, Sofia, Bulgaria; 10 Department of Vascular Medicine, Amsterdam Cardiovascular Sciences, Amsterdam UMC, University of Amsterdam, Amsterdam, Netherlands; 11 Hungarian Angioedema Center of Reference and Excellence, Semmelweis University, Budapest, Hungary; 12 Division of Allergy and Immunology, Federal University of Paraná, Curitiba, Brazil; 13 Universidad de Especialidades Espíritu Santo, Samborondón, Ecuador; 14 Respiralab Research Group, Guayaquil, Ecuador; 15 Division of Allergy and Clinical Immunology, Department of Internal Medicine, Special Hospital for Pulmonary Diseases, Zagreb, Croatia; 16 Department of Dermatology, Siriraj Hospital, Mahidol University, Bangkok, Thailand; 17 Allergy & Immunology Department, Cleveland Clinic Abu Dhabi, Abu Dhabi, United Arab Emirates; 18 Division of Allergy and Immunology, McGill University Health Centre, Montreal, QC, Canada; 19 Division of Allergy and Clinical Immunology, Erciyes University School of Medicine, Kayseri, Turkey; 20 Remedika General Hospital, Skopje, North Macedonia; 21 Romanian Hereditary Angioedema Expertise Centre, Sangeorgiu de Mures, Romania; 22 ACARE Center Sanna Clínica el Golf, San Isidro, Lima, Perú; 23 Queen Mary Hospital, University of Hong Kong, Hong Kong, Hong Kong SAR, China; 24 Hospital Universitari de Bellvitge, IDIBELL, Barcelona, Spain; 25 Department of Pediatric Respiratory Medicine, Immunology and Critical Care Medicine, Charité — Universitätsmedizin Berlin, corporate member of Freie Universität Berlin, Humboldt-Universität zu Berlin, and Berlin Institute of Health, Berlin, Germany; 26 Azienda Ospedale Università di Padova, Padova, Italy; 27 Department of Clinical Immunology and Allergology, St. Anne’s University Hospital in Brno, and Faculty of Medicine, Masaryk University, Brno, Czechia; 28 Department of Dermatology and Venerology, Kepler University Hospital, Linz, Austria; 29 Clinical Research Institute for Inflammation Medicine, Medical Faculty, Johannes Kepler University Linz, Linz, Austria; 30 Department of Dermatology, Bispebjerg Hospital, Copenhagen, Denmark; 31 Allergology Department, CSUR Angioedema (Reference Center), Hospital Universitario Virgen del Rocío, Sevilla, Spain; 32 University Saints Cyril and Methodius, Skopje, North Macedonia; 33 Centre for Clinical Immunology and Allergology, Pauls Stradins Clinical University Hospital, Center for the Diagnosis and Treatment of Allergic Diseases, Riga Stradins University, Riga, Latvia; 34 Division of Allergy and Clinical Immunology, Groote Schuur Hospital, University of Cape Town, and Allergy and Immunology Unit, UCT Lung Institute, Cape Town, South Africa

**Keywords:** angioedema, CARE, disease registry, hereditary angioedema, rare disease

## Abstract

**Background:**

The Chronic Angioedema Registry (CARE) is the first international, multicenter observational registry established to systematically collect clinical and patient-reported data from patients with recurrent angioedema of any type during routine clinical care. Here we report enrollment after 2 years of CARE, discuss challenges and present ideas to overcome them.

**Methods:**

Registry growth, recruitment yield, and distribution of angioedema diagnosis were analyzed descriptively as of December 12, 2025. Additionally, a structured web-based survey was conducted among physicians within the network of Angioedema Centers of Reference and Excellence to assess participation status, perceived barriers and facilitators, satisfaction, and communication preferences.

**Results:**

Since its inception in October 2023, CARE enrolled 550 patients across 23 centers in 18 countries [mean (range): 24 (0-302)/center, 31 (0-321)/country], achieving predefined milestones on schedule. Hereditary angioedema and mast cell–mediated angioedema were the most frequent diagnoses. The survey included 22 respondents from 18 countries, representing centers at different stages of participation. Administrative and organizational challenges, particularly ethics approval processes and limited staff resources, were identified as the main barriers. Overall satisfaction with CARE was high, increasing with advanced participation. Flexible handling of regulatory requirements, strong onboarding support, and training materials were perceived as key facilitators. Conferences, in-person meetings, and newsletters were the preferred communication channels.

**Discussion:**

CARE has rapidly evolved into a robust global real-world resource for recurrent angioedema. Addressing regulatory and organizational barriers and strengthening tailored support and communication strategies will be essential to sustain growth, enhance participation, and maximize its long-term scientific and clinical impact.

## Introduction

Angioedema (AE) is defined by transient, non-pitting swelling of the skin, mucous membranes, or deeper tissues. Recurrent forms of AE pose a diagnostic challenge, negatively impact quality of life, and certain forms are life-threatening. Most cases of recurrent AE are caused by the degranulation of mast cells (MC), i.e., AE-MC, and the subsequent release of mediators, e.g., histamine, which may come with or without wheals (hives) in (chronic) urticaria (1). More rare types of recurrent AE include bradykinin (BK) - mediated (AE-BK), drug-induced (AE-DI) or AE due to vascular endothelial dysfunction (AE-VE). AE-BK includes hereditary angioedema due to C1 inhibitor (C1INH) deficiency (HAE-C1INH), acquired AE due to C1INH deficiency (AAE-C1INH) and certain types of HAE with normal C1INH (HAE-nC1INH). In very rare cases, the exact cause for recurrent AE remains unknown (UNK), although subtypes with a positive family history (HAE-UNK) and without (AE-UNK) may exist (2).

In recent decades, many advances in the understanding and treatment of different types of recurrent angioedema have been made, including several novel-targeted therapies for both BK and MC-mediated AE (3, 4). However, robust data on disease patterns, treatment effectiveness, and disease-related quality of life (QoL) on a larger and global scale outside of controlled clinical trials, i.e., under real-life conditions, are lacking (2, 5). In addition, several novel forms of HAE have been described but with very low patient numbers and little information about natural history of disease and treatment outcomes (6, 7). Well-designed disease-specific registries, especially in rare diseases, can address gaps in clinical research and disease management with the overall aim of improving patient care.

The Chronic Angioedema Registry (CARE), commenced in October 2023, is the first international, multicenter observational disease registry for recurrent angioedema of any type. The strength of CARE is that it is a web-based disease registry which was developed by an international panel of expert physicians experienced in the treatment of various AE types. Equipped with these features, CARE collects real-life data integrated into routine clinical workflows across multiple countries and healthcare systems, enabling high-quality, standardized, longitudinal data to inform patient care, facilitate research, and support policy development (8).

After 2 years of CARE being up and running, a closer look at the progress of its distribution in specialized angioedema centers worldwide, its growth with the recruitment of new centers, the number of patients enrolled, and its implementation into clinical practice would be of help to guide future progress and the evolution of the disease registry. Additionally, deeper insights into the barriers and challenges faced during the onboarding as well as overall satisfaction and communication preferences perceived by the investigators of CARE (and those who intend to participate) would be of value. For the latter aim, a survey was initiated among the Angioedema Centers of Reference and Excellence (ACARE), which is a global network of more than 100 certified centers in more than 40 countries and 6 regions.

## Methods

### CARE registry

The detailed methods and rationale of CARE have been reported previously (8). In brief, CARE is a multicenter, observational disease registry implemented through a secure web-based platform (REDCap) hosted by the UNEV gGmbH (Germany). A structured onboarding process supports integration of new centers, and includes: signing a cooperation agreement with UNEV gGmbH, obtaining local ethics approval, and completing a training in REDCap (https://chronic-angioedema-registry.com/for-participants/).

CARE facilitates the collection of observational data during routine patient visits without altering clinical decision-making. Patients receive written information and provide informed consent before enrollment. They then complete a digital questionnaire using a smart device via a QR code interface, covering symptom burden, disease control and disease-related QoL via patient-reported outcome measures (PROMs) as well as comorbidities and response to treatments.

Physicians (investigators) subsequently complete the physician questionnaire, a structured clinical form used to confirm the patient's angioedema diagnosis and document relevant clinical information. The predominant angioedema diagnosis of each patient case documented in CARE is the responsibility of the respective CARE investigator at each center (site-reported diagnosis) and is based on the DANCE classification and nomenclature of recurrent angioedema forms as well as international guidelines for HAE and chronic urticaria (1, 2, 5). Moreover, it is possible to document other (comorbid) forms of angioedema in each patient case, which allows for the investigation of overlapping symptoms and types. In 2025, the registry was expanded by a laboratory data sheet, where investigators can optionally enter diagnostic data. There is no formal provision for a central adjudication or review of diagnoses, but this is possible through the evaluation of laboratory results and the assessment of questions regarding diagnostics and treatment response in the physician questionnaire.

Patients and physicians are asked to complete a follow up questionnaire at regular intervals, typically every 3 to 6 months related to their consultation visits.

### Survey among physicians in the ACARE network

The CARE management team, i.e., the chief scientific coordinator (TB) and the coordinator of the CARE office (AB), developed a structured web-based survey based on two years of experience with CARE including challenges discussed and documented in protocols from International Steering Committee (ISC) meetings. After a one-week pilot test phase with other investigators at ACARE Berlin, the survey was finally distributed over 5 weeks from November 12 to December 17, 2025 via social media and an email distribution list to physicians treating AE in the ACARE network (111 email addresses from 111 centers). One representative of each center was invited to participate voluntarily in the survey. Representatives from the ACARE Berlin did not participate in the survey. The full survey consisted of 41 items/questions and 5 domains, i.e., the current center's participation status (domain 1), key barriers to participation and implementation (domain 2), overall satisfaction with the registry (domain 3), communication preferences (domain 4), and perceived usefulness of proposed support measures (domain 5). (Supplementary Material). Participation status was categorized into four phases: centers that had only received information (phase 1), centers that had submitted documents to ethics with collaboration agreements in progress (phase 2), centers with ethics approval and signed agreements but not yet enrolling patients (phase 3), and centers actively enrolling patients (phase 4). Respondents from centers in phases 1–3 did not receive questions about challenges in patient enrollment (16 questions). Specific barriers and challenges as well as support needs were captured in items using rating scales from 0 to 5, with 0 corresponding to no agreement and 5 to maximum agreement. Respondents also had the option to add and rate further items. Ratings of 3 or above were considered relevant.

### Statistics

The current recruitment status of CARE was captured from REDCap, as of December 12, 2025 and summarized descriptively. For each survey question, responses were anonymized and analyzed descriptively (mean with standard deviation, respectively). The statistical analyses were performed using Microsoft ® Excel version 19.9 and SPSS (IBM SPSS statistics version 29, IBM Corporation, Armonk, NY).

## Results

### CARE—A growing global resource for clinical insights into recurrent angioedema

**Figure 1:**
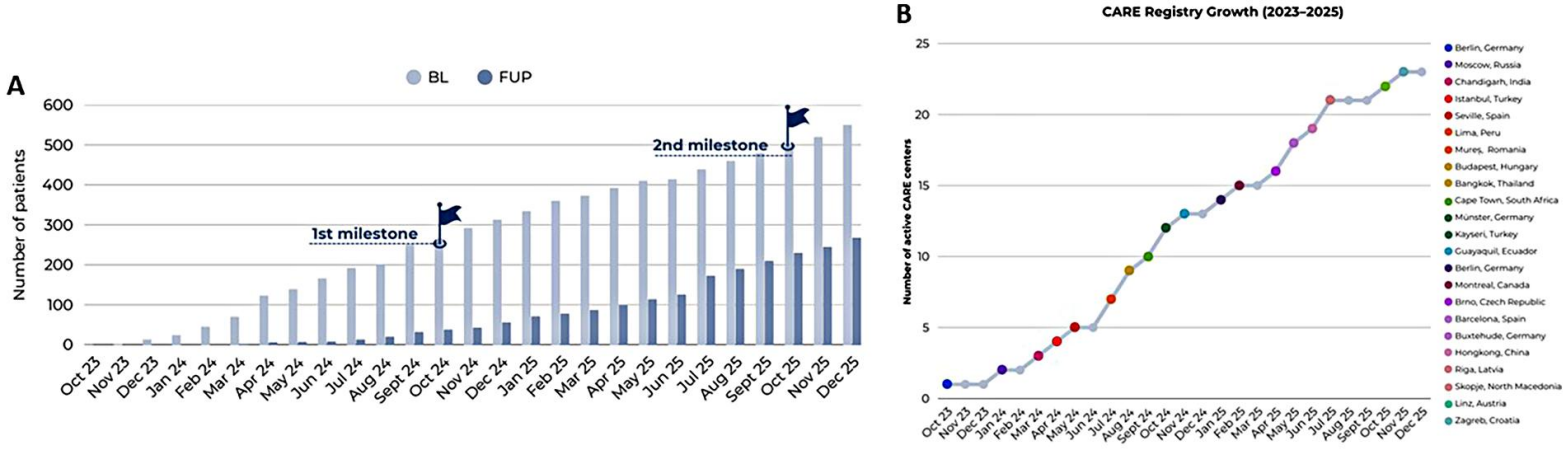
**(A)** Patient enrollment in the CARE registry as of December 12, 2025, BL = baseline entries, FUP = follow-up entries. The 1st milestone and the 2nd milestone were defined as 250 BL and 500 BL entries one and two years after the start of the registry, respectively. **(B)** Active participation of the centers in the CARE registry in the respective month.

As of December 12, 2025, the CARE registry has enrolled 550 patients (550 completed Baseline and 272 Follow up data entries) across 23 centers in 18 countries **(**Figure 1A**).** The first milestone (250 patients within the first year) and the second milestone (500 patients within two years) were achieved on schedule in October 2024 and October 2025, respectively. Most patients were recruited in the ACARE Berlin, Charité—University Medicine Berlin, Institute of Allergology (302 Baseline and 184 Follow up), followed by the ACARE Moscow City Clinical Hospital No. 52, I.M. Sechenov First, Moscow State Medical (74 Baseline and 30 Follow up) and Cape Town University of Cape Town, Department of Medicine (45 Baseline and 16 Follow up).

In total, 22 additional centers have become CARE centers since its launch in October 2023 until the end of December 2025, which corresponds to an average growth rate of 0.85 centers/month or 10 centers/year **(**Figure 1B**).** The registry continues to expand, and all physicians involved in the management of patients with recurrent angioedema are encouraged to participate.

**Figure 2:**
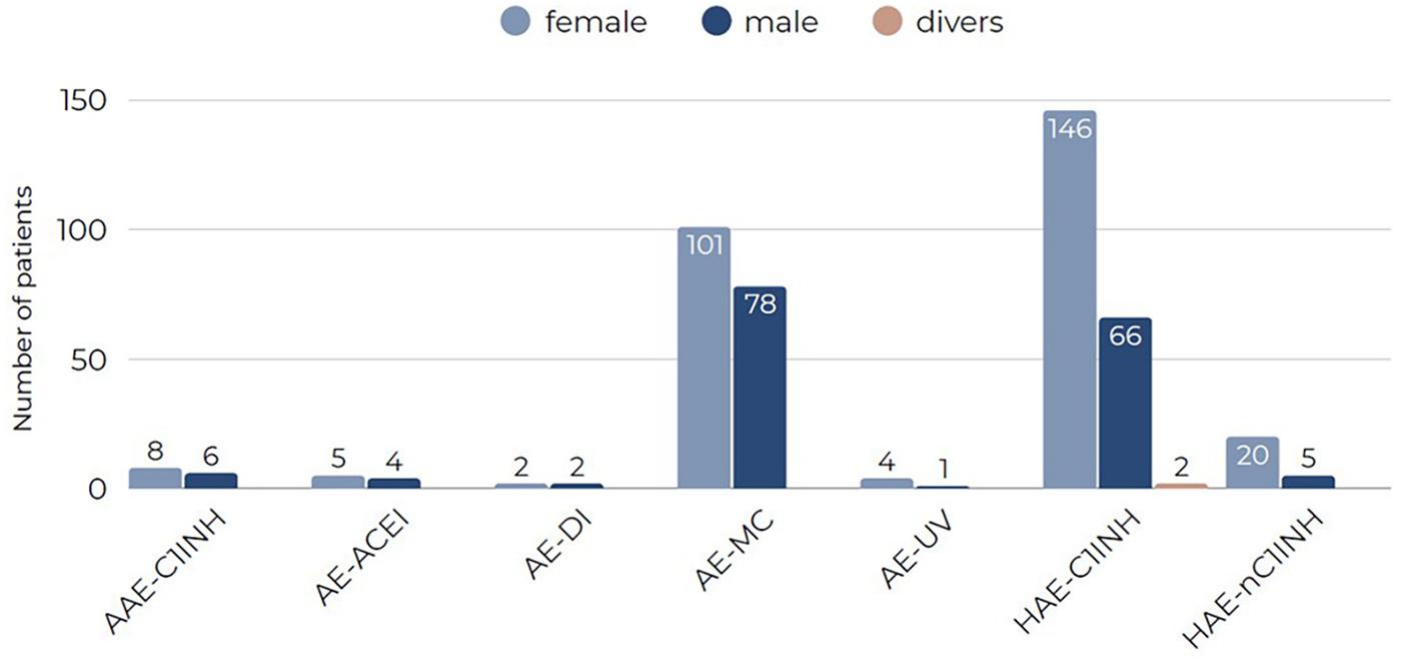
Diagnosis distribution in the CARE registry as of December 12, 2025. AAE-C1INH, acquired angioedema due to C1 inhibitor deficiency; AE-ACEI, ACE inhibitor induced angioedema; AE-DI, other drug induced angioedema; AE-MC, mast cell—mediated angioedema; AE-UV, angioedema in urticarial vasculitis; HAE-C1INH, hereditary angioedema due to C1 inhibitor deficiency; HAE-nC1INH, hereditary angioedema with normal C1 inhibitor.

The most frequent diagnosis in the CARE registry is HAE with 239 patients, among them 25 patients with confirmed diagnosis of HAE with normal C1 inhibitor (HAE-C1INH), followed by AE-MC with 179 patients. Additionally, the registry also presents 14 cases of acquired angioedema due to C1INH deficiency (AAE-C1INH). The majority of patients currently included are female **(**Figure 2**).**

### Survey participants and center status

In total, 22 respondents from 18 countries and 5 regions (Supplementary Table S1) participated in the web-based survey. Responding centers were at different stages of participation: while *n* = 8 centers were actively enrolling patients (phase 4), the majority of respondents were still in earlier phases, ranging from initial information received (phase 1, *n* = 7), documents submitted to ethics and collaboration agreement either signed or in the process of being signed (phase 2, *n* = 3), to ethics approval in place and preparation for enrollment (phase 3, *n* = 4).

### Perceived barriers and challenges for implementation of the CARE registry

**Figure 3:**
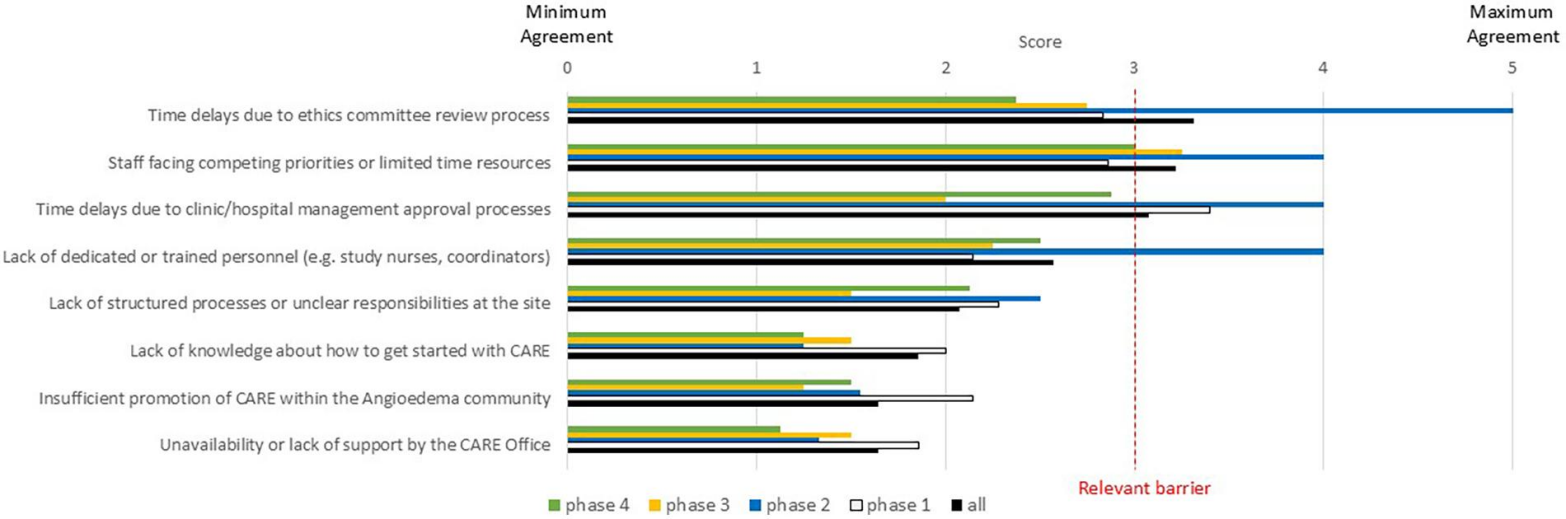
Barriers and challenges for implementation of the CARE registry in centers broken down by the respondent's participation phase (1−4). Mean scores a displayed. Phase 1: information received; phase 2: documents submitted to ethics and collaboration agreement in progress; phase 3: ethics approval received and agreement signed, but not yet enrolling patients; phase 4: actively enrolling patients. 0 = minimum agreement. 5 = maximum agreement.

Overall, administrative and organizational barriers were rated as the most relevant challenges to CARE implementation by the respondents **(**Figure 3**).** Approximately two-thirds of respondents cited the following points as significant obstacles, i.e., they rated each point with at least 3 out of 5 points: Delays due to the review process by the ethics committee (3.31 ± 1.43 points), competing priorities or limited staff time (3.2 ± 1.5 points), and delays due to approval procedures by clinic or hospital management (3.1 ± 1.5 points). A lack of dedicated or trained personnel, such as study nurses or coordinators, was also frequently reported (2.6 ± 1.55 points), whereas unclear responsibilities or insufficiently structured processes at the site were of moderate relevance (2.1 ± 1.3 points). In contrast, barriers related to awareness and support for the CARE registry were rated lower, including lack of knowledge on how to get started with CARE (1.9 ± 1.1 points), insufficient promotion within the angioedema community (1.64 ± 1.1 points), and unavailability or lack of support by the CARE Office (1.64 ± 0.9 points).

In particular, participants who stated that they were currently in the onboarding phase (*n* = 3, phase 3) reported the greatest challenges due to time delays caused by review by the ethics committee review (all 3 rated as 5 - maximum agreement) and institutional approval (all 3 rated as 4 out of 5), as well as staffing issues, i.e., time and priorities constraints and shortage of dedicated/trained personnel (4 ± 1 points each). In contrast, participants who have obtained ethical approval and were ready for inclusion at the time of the survey but have not yet recruited any patients ranked staff facing competing priorities or limited time resources (3.3 ± 1.5 points) as the highest challenge for the implementation of CARE.

### Perceived facilitators to overcome challenges for registry participation

**Figure 4:**
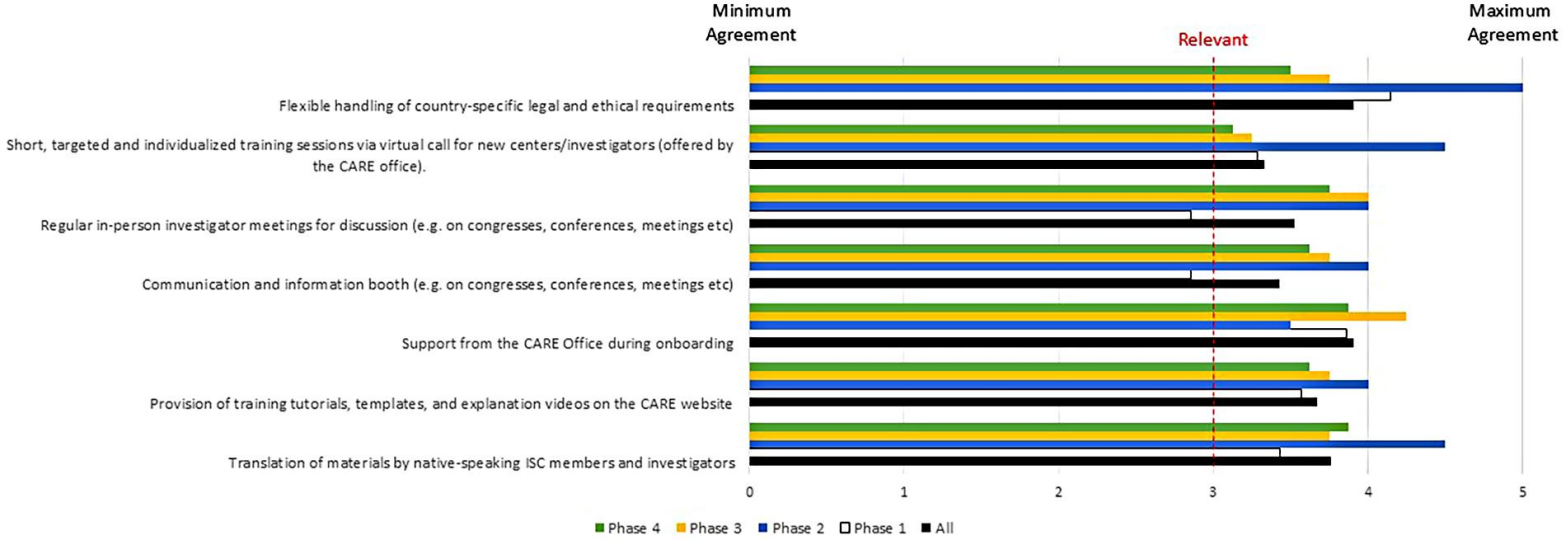
Perceived facilitators to overcome challenges for CARE registry participation broken down by the respondent's participation phase (1−4). Mean scores a displayed. Phase 1: information received; phase 2: documents submitted to ethics and collaboration agreement in progress; phase 3: ethics approval received and agreement signed, but not yet enrolling patients; phase 4: actively enrolling patients. 0 = minimum agreement. 5 = maximum agreement.

Respondents identified several support measures as particularly helpful for progress towards enrollment and data entry **(**Figure 4**).** Overall, flexible handling of country-specific legal and ethical requirements received a high rating (3.9 ± 1.4 points) and even received the highest rating (5 points) from the two centers that were in progress of getting ethics approval, emphasizing the need for adaptability within an international registry. Furthermore, strong support from the CARE Office during the onboarding process was rated highly (3.9 ± 1.5 points), especially by activated centers that had not yet recruited any patients (phase 3, *n* = 4, 4.25 ± 1.4 points). Translations of the registry content provided by native-speaking members of the CARE Steering Committee or investigators (3.8 ± 1.5 points) were also perceived highly relevant. In addition, the provision of training materials, including tutorials, templates, and videos were considered important (3.7 ± 1.5 points). One participant (phase 4) also considered how the distribution of the CARE questionnaire could be improved in the workflow of the consultation hours so that follow up questionnaires in particular could be completed before the consultation with the doctor. Such topics could be discussed, for example in regular investigator meetings in person at international congresses, which were perceived relevant (3.5 ± 1.2 points).

### Satisfaction with CARE

**Figure 5:**
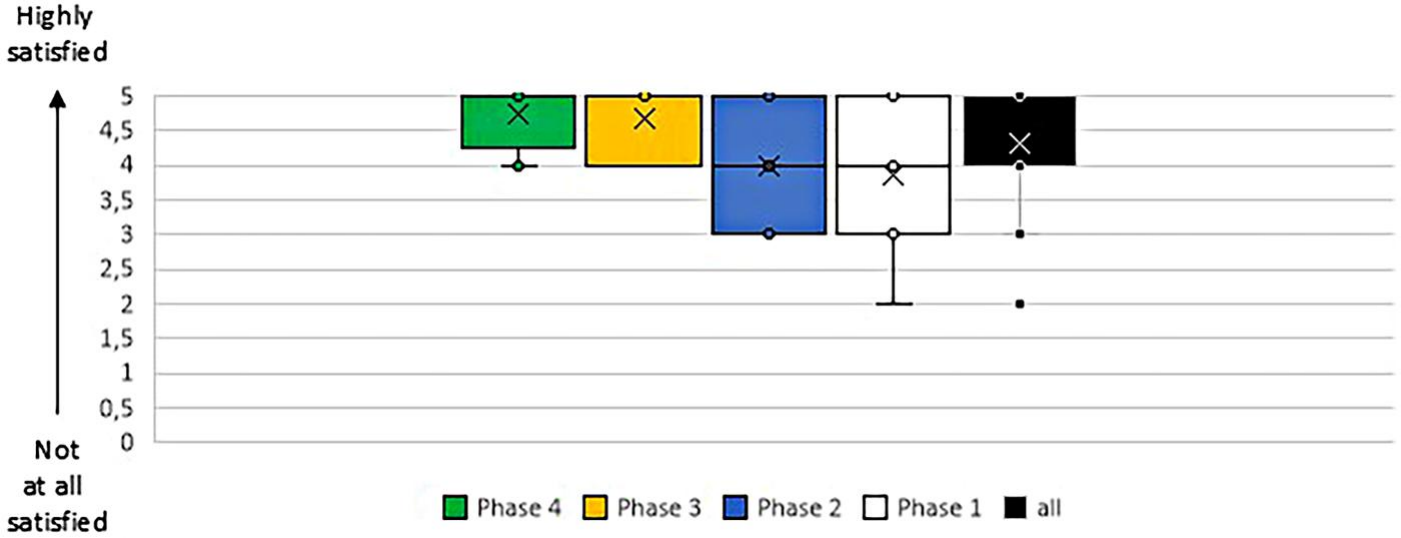
Perceived overall satisfaction with the CARE registry by the respondents broken down by the respondent's participation phase (1−4). Boxplot. Phase 1: information received; phase 2: documents submitted to ethics and collaboration agreement in progress; phase 3: ethics approval received and agreement signed, but not yet enrolling patients; phase 4: actively enrolling patients. 0 = not satisfied at all, 5 = highly satisfied.

Overall satisfaction with CARE was high, with a mean rating of 4.33 ± 0.86 points out of 5. The mean satisfaction levels increased with the progress of the onboarding process in phase 1 (3.88 ± 1.1 points) to phases 2 (4 ± 1 points) and 3 (4.7 ± 0.57 points), and were the highest among actively recruiting centers (phase 4, 4.8 ± 1.1 points) **(**Figure 5**).**

### Communication preferences

**Figure 6:**
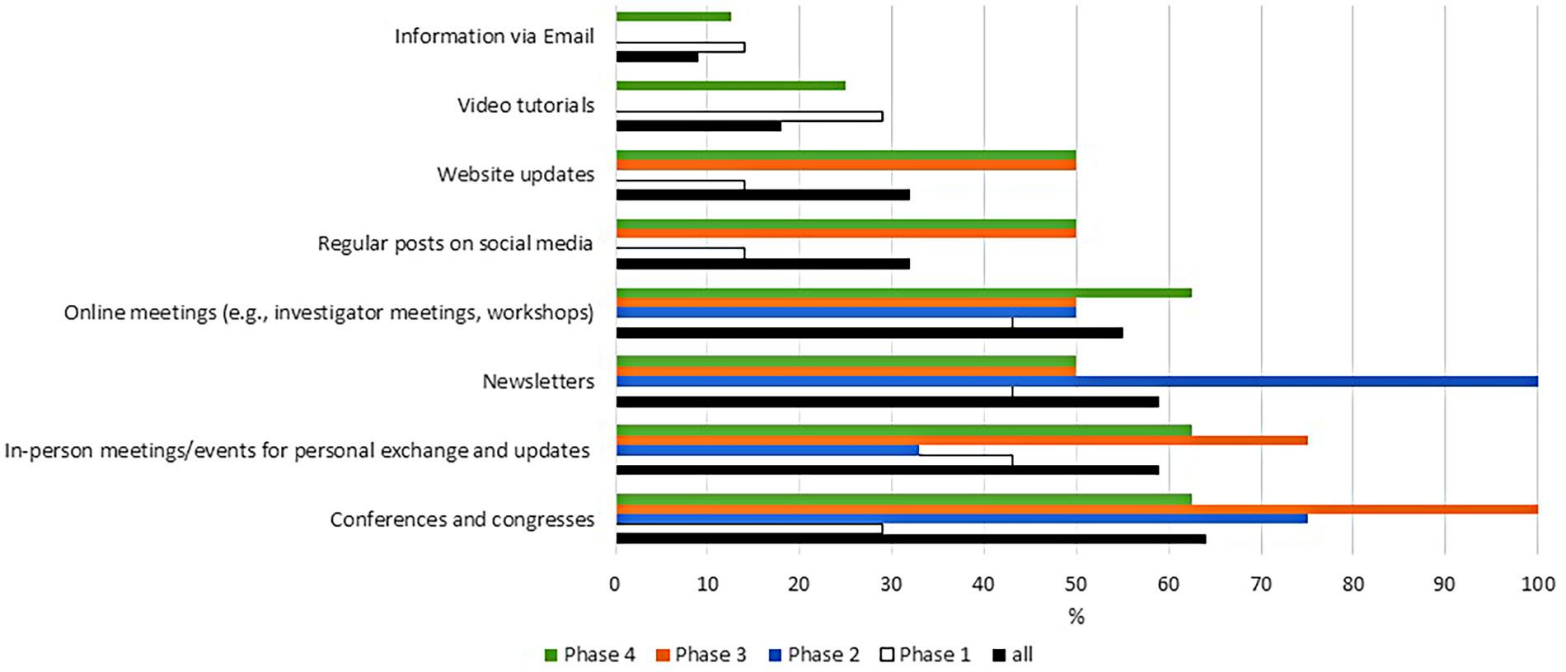
Communication preferences in the CARE registry, broken down by the respondent's participation phase (percentage of mentions). Mean scores a displayed. Phase 1: information received; phase 2: documents submitted to ethics and collaboration agreement in progress; phase 3: ethics approval received and agreement signed, but not yet enrolling patients; phase 4: actively enrolling patients.

Regarding preferred communication channels, respondents most frequently indicated events at conferences and congresses (64%, *n* = 14), in-person meetings and newsletters (57%, *n* = 13 each); Online meetings were also commonly selected (55%, *n* = 12). In contrast, regular social media posts (30%, *n* = 7 each) were reported as less useful communication tools. By far the least frequently selected options were website updates and information via email (18% and 9%, respectively) **(**Figure 6**).**

Preferences varied depending on the current participation status at CARE in the responses. While 75%, 100% and 62.5% of respondents in phase 2,3 and 4, respectively preferred communication via conferences and congresses, only 30% of respondents in phase 1 considered this channel useful. All three respondents in phase 2 preferred newsletters as a helpful tool for communication.

## Discussion

The recruitment success and global expansion of CARE over the past two years indicate significant progress in the development of a robust, global resource for clinical insights into all types of recurrent AE. The timely achievement of the planned recruitment milestones — 250 patients within the first year and 500 patients within two years — demonstrates the feasibility of successfully enrolling a substantial cohort within a relatively short period. The registry's continued growth, with 550 patients enrolled across 23 centers in 18 countries as of December 2025, underscores its broad international appeal and the increasing importance of collaborative data collection in rare disease research. The increasing number of newly participating centers, particularly those still in early phases of onboarding, highlights sustained global interest in CARE, while also emphasizing the need for continued engagement strategies to further improve representativeness and recruitment yield.

The observed diagnostic distribution among enrolled patients illustrates the marked heterogeneity of recurrent AE and provides a solid foundation for future comparative and subtype-specific analyses within CARE. In particular, the inclusion of patients with HAE-nC1INH and AAE-C1INH represents a key strength of the registry, as these rare and heterogeneous subgroups remain insufficiently characterized in clinical trials. The higher number of patients with HAE in CARE compared with the more common AE-MC likely reflects registry and referral dynamics. As HAE is a rare disease, it typically attracts greater clinical and research interest, leading to closer patient monitoring and more structured long-term follow-up in specialized expert centers. In contrast, AE-MC is managed in many countries in primary care or general allergy/dermatology settings, may not be systematically referred to participating centers, and represents a more heterogeneous phenotype, which can reduce motivation and feasibility for routine registry inclusion. Not least, AE-MC is also captured in the Chronic Urticaria Registry (CURE), the partner registry of CARE, so centers participating in both registries may prefer to include patients there (9).

The disproportionate representation of female patients in CARE, particularly for patients with HAE, could be explained, on the one hand, by greater use of healthcare services and higher participation rates in research (10–12). On the other hand, disease expression might be more pronounced in women, as hormonal factors (especially estrogen exposure) can exacerbate attack frequency and severity (13, 14). This effect is even more evident in HAE-nC1INH (15), which has been reported predominantly in female patients and may further contribute to the female-skewed distribution observed in the registry.

At present, patient recruitment is unevenly distributed across centers and countries, with Germany — particularly the ACARE Berlin — contributing a large proportion of enrolled patients. This likely reflects both the early involvement and coordinating role of the ACARE Berlin in the development and management of CARE, as well as differing local capacities and barriers to registry implementation in other regions. These imbalances are not unexpected in the early phases of a global registry but underline the importance of understanding and addressing center-specific challenges. This is probably influenced by staff workload and limited time for patient recruitment and data entry, as well as differences in country size and population. In addition, within the time constraints of a standard consultation, there may be insufficient time for patients to complete questionnaires, as this requires additional time beyond routine clinical care.

The accompanying survey among ACARE centers provides valuable insights into barriers, facilitators, and user perceptions related to CARE implementation. Overall satisfaction with CARE was high, confirming its perceived scientific and clinical value. Importantly, satisfaction increased progressively with advancing stages of participation and was highest among centers actively recruiting patients. This gradient suggests that increasing hands-on experience with CARE is associated with more favorable perceptions of usability and benefit, supporting the notion that the registry is perceived as a practical and user-friendly tool once integrated into routine clinical workflows. Such perceptions are critical for the long-term sustainability of any registry, as they directly influence continued engagement and data completeness.

Administrative and organizational challenges in participating country level — particularly delays related to ethics committee reviews and institutional approval processes — were identified as the most relevant barriers to participation. Competing clinical priorities and limited staff resources further complicated implementation, especially in centers with limited research infrastructure. In contrast, barriers related to awareness of CARE, clarity of processes, or support from the CARE Office were rated comparatively low, indicating that the central coordination and support structures are functioning effectively. These findings highlight that many of the key obstacles lie outside the direct control of the CARE management team itself and are inherent to multinational research initiatives in rare diseases. Some surveys and evaluations of rare disease registries conducted in other settings have reported similar barriers and facilitators to those identified for CARE, lending external validity to our findings. For example, a survey conducted among registry leaders for multiple rare diseases highlighted the importance of governance structures, data quality, and sustainable management systems as essential registry features, with registries often struggling with long-term sustainability and standardized processes across sites (16). In particular, the global registry for HAE-C1INH highlighted challenges related to long-term sustainability, uneven patient distribution and variability in data completeness across participating sites (17).

At the same time, the survey results clearly point to actionable facilitators that can mitigate these challenges. Flexibility in accommodating country-specific legal and ethical requirements, strong onboarding support, provision of training materials, and availability of translations were consistently rated as highly relevant. Based on these insights, the CARE management team has already initiated — and will continue to implement — targeted measures to further facilitate center recruitment and, in particular, patient enrollment. These include streamlining onboarding procedures, expanding tailored training resources, enhancing workflow integration of patient questionnaires by regular in-person meetings and webinars, and intensifying direct support during the early recruitment phase. Such adaptive strategies are essential to lower participation thresholds and accelerate patient inclusion across diverse healthcare settings.

The preference for in-person meetings, such as those at international congresses and conferences, underscores the continued importance of personal interaction in fostering collaboration and knowledge exchange as well as mutual support and motivation. Online meetings, although slightly less preferred, were also seen as valuable, highlighting the evolving role of digital communication tools in maintaining engagement across a geographically dispersed network. In contrast, email communication was consistently rated as the least helpful channel, suggesting that passive or asynchronous updates alone may be insufficient to engage centers and investigators effectively. The usefulness of newsletters and other digital tools, such as website updates and video tutorials, further supports the registry's hybrid communication strategy, blending traditional in-person meetings with targeted digital resources for broader accessibility.

The findings of the survey should be interpreted in the context of several limitations. First, the low response rate for the survey relative to all invited centers (22 centers of 111 invited centers responded, i.e., 20%) may limit the generalizability of the results. Only 36% of centers actively recruiting patients into CARE participated in the survey, whereas 45% of respondents were still in the onboarding phase. Second, centers joined CARE at different time points and experienced varying onboarding procedures as documentation and entry requirements evolved over time. However, due to the small number of respondents, we were unable to compare responses between early-joining centers and those enrolled later, which may have introduced selection and recall bias. The challenges may also differ by center status—while fully onboarded centers are more likely constrained by the practical workload of patient recruitment and data entry, centers still in the onboarding phase may be primarily delayed by pending ethics approval. And third, the cross-sectional design of the survey captures perceptions at a single time point and does not reflect changes over time. Longitudinal assessments of center participation and feedback would be valuable to provide a more comprehensive understanding of the registry's evolution.

In conclusion, CARE has demonstrated rapid growth, high user satisfaction, and increasing international adoption within its first two years. By systematically addressing identified barriers and continuously adapting support measures based on user feedback, CARE is well positioned to further expand its global footprint and scientific impact. Importantly, the insights gained regarding the challenges and barriers to participation, as well as the strategies identified to overcome at least some of them, may also serve as a valuable guide for future initiatives aimed at establishing and managing global disease registries. These findings support CARE's potential to become a cornerstone for real-world research in recurrent AE and provide a valuable framework for the development of future global registries in rare diseases.

## Data Availability

The original contributions presented in the study are included in the article/Supplementary Material, further inquiries can be directed to the corresponding author.

## Supplementary material

Supplementary Table S1 Countries and Regions of respondents to the survey.
