# Supplementary material for: The Chronic Angioedema Registry: what the first 2 years since the implementation of the global registry have taught us
Source: Front Allergy. 2026 Jun 22;7:1799211. doi: 10.3389/falgy.2026.1799211 (PMC13333679; doi:10.3389/falgy.2026.1799211)
Supplement: Supplementary file 1 [file Datasheet1.pdf]

CARE Registry

Key Implementation Challenges

Please review the list below on potential challenges in setting up and enrolling patients for the Chronic Angioedema Registry. Your feedback will help identify common barriers and support shared learning within the network.

Please enter your affiliation.
(We kindly request this information to identify the center's principal investigator and to discuss with him or her who should be included as a co-author representing the center.)

Please enter your city.

Current status of your center

Please let us know which phase your center is currently in.

- ◯ **Phase 1**: Information received; no submission yet.
- ◯ **Phase 2**: Submitted to ethics; collaboration agreement either signed or in the process of being signed.
- ◯ **Phase 3**: Ethics approval obtained and collaboration agreement signed. **NOT** enrolling patients yet.
- ◯ **Phase 4**: Ethics approval obtained and collaboration agreement signed. Actively enrolling patients.

Set up phase

Please rate the following setup phase challenges based on your experience, using a scale from 1 to 5, where 1 means 'does not apply at all' and 5 means 'fully applies'.

Time delays due to ethics committee review process.

1	2	3	4	5

Time delays due to clinic/hospital management approval processes.

1	2	3	4	5

Lack of dedicated or trained personnel (e.g. study nurses, coordinators).

1	2	3	4	5

Staff facing competing priorities or limited time resources.

1	2	3	4	5

Lack of structured processes or unclear responsibilities at the site.

1	2	3	4	5

Unavailability or lack of support by the CARE Office.

1	2	3	4	5

Lack of knowledge about how to get started with CARE.

1	2	3	4	5

Insufficient promotion of CARE within the Angioedema community.

1	2	3	4	5

Other 1, please specify

○ Sonstiges

Please rate 'other 1', if you’ve made any additions.

1	2	3	4	5

Other 2, please specify

- ◯ Sonstiges

Please rate 'other 2', if you've made any additions.

1	2	3	4	5

Other 3, please specify

- ◯ Sonstiges

Please rate 'other 3', if you've made any additions.

1	2	3	4	5

Set up phase

Please rate the following setup phase challenges based on your experience, using a scale from 1 to 5, where 1 means 'does not apply at all' and 5 means 'fully applies.'

Time delays due to ethics committee review process.

1	2	3	4	5

Time delays due to clinic/hospital management approval processes.

1	2	3	4	5

Lack of dedicated or trained personnel (e.g. study nurses, coordinators).

1	2	3	4	5

Staff facing competing priorities or limited time resources.

1	2	3	4	5

Lack of structured processes or unclear responsibilities at the site.

1	2	3	4	5

Unavailability or lack of support by the CARE Office.

1	2	3	4	5

Lack of knowledge about how to get started with CARE.

1	2	3	4	5

Insufficient promotion of CARE within the community.

1	2	3	4	5

Other 1, please specify

○ Sonstiges

Please rate 'other 1', if you’ve made any additions.

1	2	3	4	5

Other 2, please specify

◯ Sonstiges

Please rate 'other 2', if you’ve made any additions.

1	2	3	4	5

Other 3, please specify

◯ Sonstiges

Please rate 'other 3', if you’ve made any additions.

1	2	3	4	5

Inclusion of patients

Please rate the following items based on your experience, using a scale from 1 to 5, where 1 means 'does not apply at all' and 5 means 'fully applies.'

Lack of dedicated or trained personnel (e.g. study nurses, coordinators).

1 2 3 4 5

Staff facing competing priorities or limited time resources.

1 2 3 4 5

Lack of structured processes or unclear responsibilities at the site.

1 2 3 4 5

Difficulties in patient communication or engagement (e.g., low participation, lack of motivation).

1 2 3 4 5

Patient-related technical barriers (e.g. lack of smartphone or internet access, insufficient digital skills)

1 2 3 4 5

Insufficient motivation from the CARE management team

1 2 3 4 5

Site staff-related technical barriers to manage the registry (e.g. RedCap)

1 2 3 4 5

High burden for patients (e.g. questionnaires perceived as too long or time-consuming)

1 2 3 4 5

Difficulty in identifying suitable patients

1 2 3 4 5

Questions are unclear or not easily understood by patients

1	2	3	4	5

Other 1, please specify:

◯ Sonstiges

Please rate 'other 1', if you’ve made any additions.

1	2	3	4	5

Other 2, please specify:

◯ Sonstiges

Please rate 'other 2', if you’ve made any additions.

1	2	3	4	5

Other 3, please specify:

◯ Sonstiges

Please rate 'other 3', if you’ve made any additions.

1	2	3	4	5

Feedback on implemented measures to improve the management and use in CARE

Please rate how helpful each of the following actions could be/had been in addressing the related challenges, using a scale from 1 to 5, where 1 means 'not helpful at all' and 5 means 'very helpful.'

Translation of materials by native-speaking ISC members and investigators.

1	2	3	4	5

Provision of training tutorials, templates, and explanation videos on the CARE website.

1	2	3	4	5

Support from the CARE Office during onboarding.

1	2	3	4	5

Communication and information booth on congresses like Global Angioedema Forum (GAF).

1	2	3	4	5

Regular in-person investigator meetings for discussion on congresses e.g. GAF

1	2	3	4	5

Flexible handling of country-specific legal and ethical requirements.

1	2	3	4	5

Short, targeted and individualized training sessions via virtual call for new centers/investigators (offered by the CARE office).

1	2	3	4	5

Other ideas for measures that could further improve the CARE registry?

○ Sonstiges

Overall Satisfaction

How satisfied are you with CARE?

☆ ☆ ☆ ☆ ☆

Information and Communication Preferences

What is the best way to keep you informed about CARE updates? (Please choose all that apply.)

- ☐ Conferences and congresses
- ☐ In-person meetings and events for personal exchange and updates (e.g., investigator meetings, workshops)
- ☐ Online meetings (e.g., investigator meetings, workshops)
- ☐ Newsletters
- ☐ Regular posts on social media
- ☐ Website updates
- ☐ Video tutorials
- ☐ Sonstiges

Dieser Inhalt wurde von Microsoft weder erstellt noch gebilligt. Die von Ihnen übermittelten Daten werden an den Formulareigentümer gesendet.

Microsoft Forms
