# Supplementary material for: The Chronic Angioedema Registry: what the first 2 years since the implementation of the global registry have taught us
Source: Front Allergy. 2026 Jun 22;7:1799211. doi: 10.3389/falgy.2026.1799211 (PMC13333679; doi:10.3389/falgy.2026.1799211)
Supplement: Supplementary Table S1 — Countries and Regions of respondents to the survey. [file Supplementaryfile1.pptx]

## Slide 1
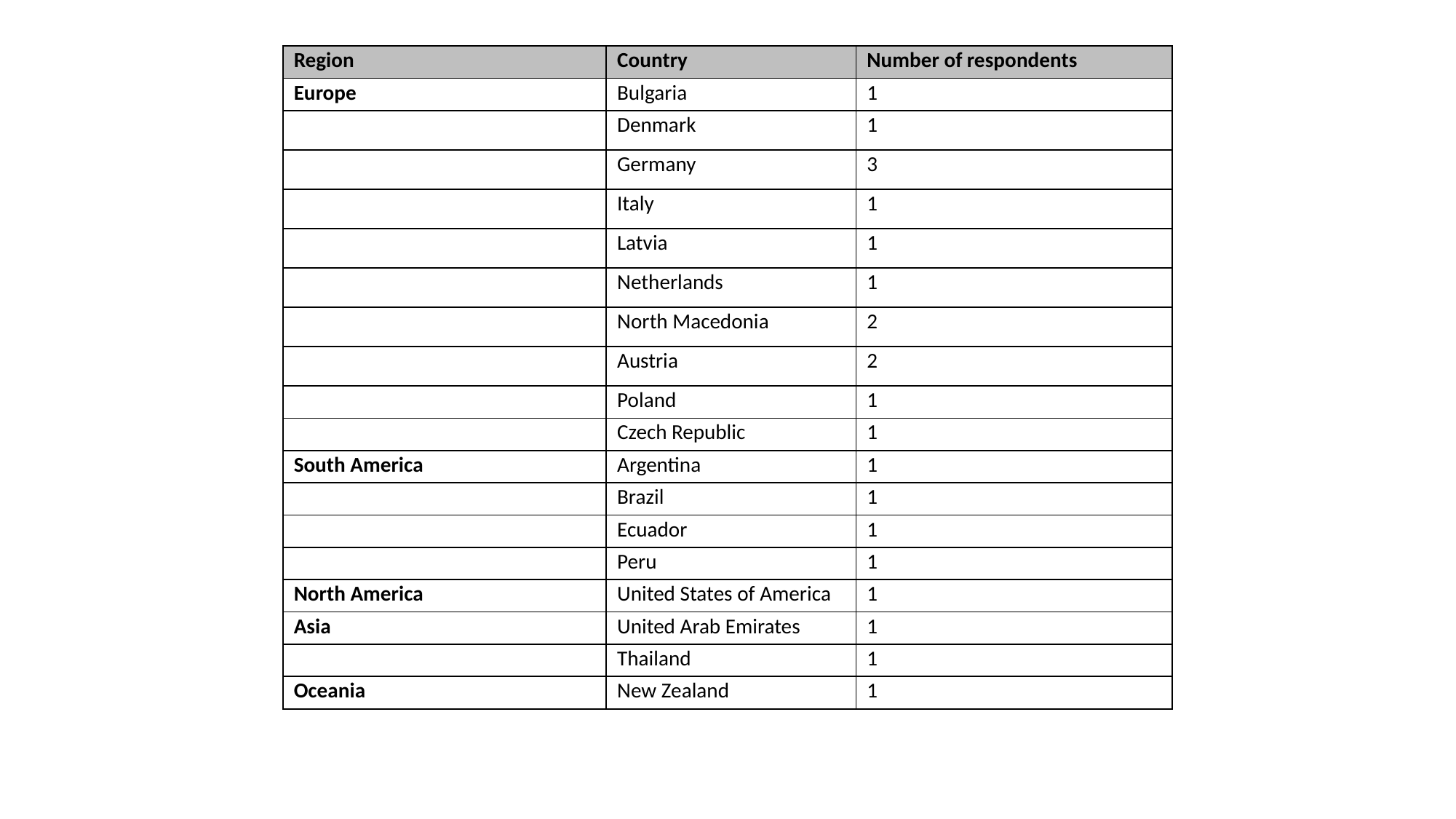

| Region | Country | Number of respondents |
| --- | --- | --- |
| Europe | Bulgaria | 1 |
| | Denmark | 1 |
| | Germany | 3 |
| | Italy | 1 |
| | Latvia | 1 |
| | Netherlands | 1 |
| | North Macedonia | 2 |
| | Austria | 2 |
| | Poland | 1 |
| | Czech Republic | 1 |
| South America | Argentina | 1 |
| | Brazil | 1 |
| | Ecuador | 1 |
| | Peru | 1 |
| North America | United States of America | 1 |
| Asia | United Arab Emirates | 1 |
| | Thailand | 1 |
| Oceania | New Zealand | 1 |
